# Comparison of Extended-Spectrum β-Lactamase (ESBL) CTX-M Genotypes in Franklin Gulls from Canada and Chile

**DOI:** 10.1371/journal.pone.0141315

**Published:** 2015-10-23

**Authors:** Jonas Bonnedahl, Johan Stedt, Jonas Waldenström, Lovisa Svensson, Mirva Drobni, Björn Olsen

**Affiliations:** 1 Centre for Ecology and Evolution in Microbial Model Systems, School of Natural Sciences, Linnaeus University, SE - 391 82 Kalmar, Sweden; 2 Department of Infectious Diseases, Kalmar County Hospital, SE-391 85 Kalmar, Sweden; 3 Department of Medical Sciences, Uppsala University, SE-751 85 Uppsala, Sweden; US Geological Survey, UNITED STATES

## Abstract

Migratory birds have been suggested to contribute to long-distance dispersal of antimicrobial resistant bacteria, but tests of this hypothesis are lacking. In this study we determined resistance profiles and genotypes of ESBL-producing bacteria in randomly selected *Escherichia coli* from Franklin´s gulls (*Leucophaeus pipixcan*) at breeding sites in Canada and compared with similar data from the gulls' wintering grounds in Chile. Resistant *E*. *coli* phenotypes were common, most notably to ampicillin (30.1%) and cefadroxil (15.1%). Furthermore, 17.0% of the gulls in Canada carried ESBL producing bacteria, which is higher than reported from human datasets from the same country. However, compared to gulls sampled in Chile (30.1%) the prevalence of ESBL was much lower. The dominant ESBL variants in Canada were *bla*_CTX-M-14_ and *bla*_CTX-M-15_ and differed in proportions to the data from Chile. We hypothesize that the observed differences in ESBL variants are more likely linked to recent exposure to bacteria from anthropogenic sources, suggesting high local dissemination of resistant bacteria both at breeding and non-breeding times rather than a significant trans-hemispheric exchange through migrating birds.

## Introduction

*Enterobacteriacae* with extended-spectrum β-lactamase (ESBL) have increased in frequency in both patients and domestic animals during the last decades, and are now considered as an escalating challenge for global health care [1]. This increase is not due to clonal expansion of single genotypes, as there is considerable variation in ESBL genotype distribution between different geographical regions [1–4]. The rapid spread of ESBLs has largely consisted of different CTX-M types, but the mechanisms behind its greater penetration into *E*. *coli* populations compared to other ESBL classes is largely unknown [4].

At the continental level, South America has the highest reported ESBL levels [1, 5], while North America has the lowest levels, especially for CTX-M variants [1]. However, the situation is changing and in recent years CTX-M variants have increased in prevalence in the US, strongly manifested with spread of the *E*. *coli* ST131 with the *bla*_CTX-M 15_ ESBL genotype [6]. In Canada, CTX-M variants have been more widespread. For instance, a survey conducted in 2009 covering 8 of the 10 Canadian provinces found an average ESBL prevalence of 4.3% in *E*. *coli* isolated from patients attending hospitals (clinics, emergency rooms, medical and surgical wards, and intensive care units) [7, 8]. In this Canadian material the *bla*_CTX-M-15_ genotype is most common, and ESBLs was predominantly detected in at least 7 wide-spread *E*. *coli* sequence types, including ST131 and members of the ST10 clonal complex [7, 9].

In Latin America ESBL-producing *E*. *coli* are frequently recovered, with prevalence rates ranging from 8.5% in blood samples, to 18.1% in samples from multiple patient sources and reaching 45–51% in some hospital *Klebsiella* populations [1]. The high levels of ESBL-producing bacteria in countries south of North America constitutes a potential threat for dissemination of ESBLs northwards to the US and Canada through movement of people, animals and goods [10]. Additionally, but less investigated, large numbers of migratory birds that winter in South America return to their breeding grounds in North America each spring, potentially carrying ESBL-producing bacteria in their intestines. The majority of these birds are songbirds, and likely of limited importance for bacterial transmission to man since they seems to carry low levels of resistant bacteria [11–13]. However, the migratory avifauna also includes larger birds, and birds that occur in proximity to humans and domestic animals, thereby potentially allowing for dissemination of resistant bacteria between sources. One such bird is the Franklin's gull (*Leucophaeus pipixcan*), that breeds in freshwater marshes on the prairies in the northern part of the US and south Canada [14]. Franklin's gulls spend the non-breeding period along the Pacific coast in Chile and Peru, often occurring in very high densities at river outlets [14].

Recently, a study of antibiotic resistance in *Enterobacteriacae* from Franklin's gulls in Chile showed that 30.1% of the gulls carried ESBL, including genotypes that are of importance for human health [15]. Such high levels of resistant bacterial phenotypes in this migratory bird species at the wintering grounds, and the dense colonies in a restricted area of North America during breeding times, allow for testing the hypothesis that wild birds can bring resistant bacteria during transcontinental migration. To assess this, we collected fecal samples from Franklin's gulls in Winnipeg, Canada, in an area with many large breeding colonies, and compared prevalence and genetic profiles of ESBL, with focus on CTX-M, to Franklin's gulls sampled on wintering grounds in Chile [15].

## Ethical statement

A written permission to perform the gull sampling on the site visited was issued from the Canadian wildlife service. Since the fecal samples were taken from the ground there was no interaction with the gulls at all.

## Material and Methods

### Sampling

Field sampling was conducted in mid-June 2010 in Winnipeg, Canada, during the early part of the gulls' breeding season. The Franklin's gull breeding colonies were inaccessible, as they were located in large prairie marshes with restricted access. Thus, instead of sampling the birds at their nests, we sampled Franklin's gull feces at the Brady Road Landfill, a site that many gulls use for foraging. Fresh droppings were collected on the ground where gulls were roosting. To ascertain that the samples were fresh, roosting flocks of gulls were chased off by the field staff, and only fresh deposited spats collected. In order to avoid collecting more than one sample per bird, only large flocks were chosen and the number of collected samples per flock was always significantly lower than the estimated number of individuals in the flock. Samples were collected using sterile cotton swabs which were immediately inoculated into a bacterial freeze medium (Luria broth; BD, Sparks, USA, phosphate buffered saline containing 0.45% Nacitrate, 0.1% MgSO_4_, 1% (NH_4_)_2_SO_4_, and 4.4% glycerol). Directly after sampling, the samples were transported to the University of Manitoba, where they were stored in -80°C before sent by courier to Sweden on dry ice. The sampled gulls most likely breed north of the Winnipeg city in the Oak Hammock Marsh.

### *E*. *coli* isolation and antibiotic resistance testing

From each sample, one randomly chosen *E*. *coli* was tested for susceptibility to 10 different antibiotic agents using standard methods [16]. The agents used were selected to represent commonly used agents for *E*. *coli* infections in human and veterinary medicine: ampicillin 10 μg/disc, cefadroxil 30 μg/disc, chloramphenicol 30 μg/disc, nalidixic acid 30 μg/disc, nitrofurantoin 100 μg/disc, mecillinam 10 μg/disc, tetracycline 30 μg/disc, tigecycline 15 μg/disc, streptomycin 10 μg/disc and trimethoprim/sulfamethoxazole 25 μg/disc (all antibiotics from Oxoid Ltd, Cambridge, UK). As of yet, there is no EUCAST recommendations for susceptibility determination of streptomycin in *E*. *coli* and we therefore used the methods suggested by Kronvall, Kahlmeter [17] to set susceptibility cut-off for this compound.

*E*. *coli* with resistant phenotypes to cefadroxil but with no phenotypic signs of ESBL production (no synergy with clavulanic acid) were subjected to AmpC phenotype testing by antibiotic disc diffusion on Mueller-Hinton agar using the AmpC confirmation test (Rosco Diagnostica, Taastrup, Denmark).

### Isolation and characterization of ESBL-producing bacteria

In total, 400 samples were collected during 4 field days. Out of these, 364 gave bacterial growth in the laboratory. Initially, all samples were enriched using brain heart infusion broth (Becton Dickinson, Franklin Lakes, NJ, USA) supplemented with 16 mg/L vancomycin, for 18–24 h in 37°C. Samples were then inoculated on chromID^TM^ ESBL plates (bioMérieux, Solna, Sweden), according to the manufacturer’s instructions. From ESBL plates with growth of more than one bacterial species, each presumable species was chosen. Colonies were isolated and species identity confirmed by biochemical testing, and in some cases validated by MALDI/TOF analysis. ESBL-production was confirmed with the cefpodoxime/cefpodoxime+clavulanic acid double-disc test in accordance with the manufacturer’s instructions (MAST Diagnostics, Bootle, UK).

From positive samples, the *bla*_CTX-M_ genes were detected using a multiplex real-time PCR protocol [18] on a StepOnePlus real-time PCR machine (Applied biosystems). This detection method includes four different hydrolysis probes, each specific for a specific CTX-M group (CTX-M-1, CTX-M-2, CTX-M-9, and CTX-M-8/-25) [18]. PCR-positive isolates were sequenced using specific primers and protocols [19, 20] and a commercial sequence service (Eurofins MWG Operon, Ebersberg, Germany). The presence of *bla*_TEM_ and *bla*_SHV_ genes was detected using the primers developed by Pitout, Thomson [21] and a SYBR Green-based real-time PCR protocol [22], and *bla*_TEM_ and *bla*_SHV_ positive isolates were sequenced using the amplification primers. *E*. *coli* ATCC 25922 (SHV- and TEM-negative), *Klebsiella pneumoniae* ATCC 700603 (SHV-positive, TEM-negative) and *E*. *coli UKNE7630* (TEM-positive, SHV-negative) were used as control strains.

Twenty randomly chosen ESBL-producing *E*. *coli* were characterized with multilocus sequence typing (MLST) using the scheme of Wirth et al. (2006). The allele designations were determined via an online database (http://mlst.ucc.ie/mlst/dbs/Ecoli) and novel ST designations were provided by the database curator.

### Reference material

As reference dataset we used Franklin's gull data from a recent study from our research group. This material was collected in Chile and consisted of 267 *E*. *coli* with determined resistance profiles and 112 genotyped ESBL-producing *E*. *coli* isolated from gulls (Table 1) [15]. In addition, the Chilean material included 49 human fecal samples where 6 (12.2%) ESBL was isolated. When *E*. *coli* strains (both carrying ESBL and not carrying ESBL) were sequenced typed there was a large variation in STs. In total there were 23 new STs found in gull *E*. *coli* and eight in human *E*. *coli*. The most common ST type in both sources was ST10 but there were a diverse population when phylogenetic analysis was performed.

**Table 1:** The distribution of ESBL variants (CTX-M, SHV and TEM) in ESBL-producing bacteria isolated from Franklin's gulls in Canada and Chile [15].

Canada			
*E*. *coli* (n)	CTX-M	SHV	TEM
2	CTX-M-1	-	-
18	CTX-M-14	-	-
6	CTX-M-14	-	+
13	CTX-M-15	-	-
3	CTX-M-15	-	+
1	CTX-M-3	-	-
1	CTX-M-55	-	+
8	-	+	-
*Kl*. *phenomena* (n)			
1	CTX-M-14	+	-
1	CTX-M-15	-	+
2	CTX-M-15	+	+
2	-	+	-
2	-	+	+
Chile			
*E*. *coli* (n)			
101	CTX-M-1	-	-
19	CTX-M-1	-	+
2	CTX-M-1	+	-
2	CTX-M-9	-	-
2	CTX-M-9	-	+
1	-	+	-

## Results

### General resistance

In total 174 *E*. *coli* were isolated from 364 gull samples. These isolates were tested to a panel of 10 antibiotics and 72 (47.4%) isolates were resistant to ≥1 agent, and 16 (9.2%) of the isolates were resistant to ≥3 agents (Table 1). The highest levels of resistance were found to ampicillin (30.1%) and cefadroxil (15.1%), while no isolate was resistant to nitrofurantoin (Table 2). In cases where the sample sizes permitted, the resistance levels were compared between gull isolates from Canada and Chile. The only statistically significant differences were seen for ampicillin (p>0.000) and cefadroxil (p>0.000), both of which had higher levels in Canada (Fisher’s exact test; Table 1). Numerically, the greatest difference in resistance level was observed to cefadroxil, where 15.1% of the Canadian gull *E*. *coli* was resistant compared to only 1.1% of the Chilean gull *E*. *coli*.

**Table 2:** The number of *E*. *coli* (percent in brackets) with phenotypic resistance to ten antibiotics isolated from Franklin's gulls in Canada and Chile.

Antibiotic	Canada n (%)	Chile n (%)	Fisher's exact test
Nalidixic acid	10 (5.7)	18(6.7)	P = 0.84
Streptomycin	14 (8)	16 (6)	P = 0.17
Tetracycline	20 (11.5)	22 (8.2)	P = 0.32
Ampicillin	66 (37.9)	27 (10.1)	P<0.000
Chloramphenicol	2 (1.1)	6 (2.2)	P = 0.49
Cefadroxil	26 (14.9)	3 (1.1)	P<0.000
Tigecyline	1 (0.6)	1 (0.4)	P = 1.0
Nitrofurantoin	0 (0)	0 (0)	-
Mecillinam	2 (1.1)	3 (1.1)	P = 1.0
Trimethoprim–Sulphamethoxazole	7 (4)	10 (3.7)	P = 1.0

### AmpC phenotypes

Each randomely chosen *E*. *coli* carrying resistant phenotype to cefadroxil were tested for AmpC production. All isolates were in this analyse positive for AmpC and negative for ESBL which makes us suggest that the AmpC prevalence were high in the material. However, since this was not the scoop in this article no further analysis were performed.

### ESBL genotypes

In total 62 ESBL were isolated, including two samples that contained two different ESBL carrying bacterial species. Of these 55 were *E*. *coli* and seven *Klebsiella pneumoniae*, together representing 17.0% of the total samples with bacterial growth. Genotyping identified CTX-M in 51 samples, SHV in 13 samples and TEM in one sample (Table 1).

All CTX-M positive samples belonged to the CTX-M 1 group (*bla*_CTX-M-1,_ *bla*_CTX-M-3,_ *bla*_CTX-M-15,_ *bla*_CTX-M-55_) or CTX-M 9 (all bla_CTX-M-14_). Five SHV variants (*bla*_shv-2,_ *bla*_shv-2A,_ *bla*_shv-11_ *bla*_shv-12_ *bla*_shv-14_) and one TEM (bla_TEM-52_) were identified (Table 2). Further, 12 *bla*_TEM-1_ and one *bla*_SHV-1_ were identified on the ESBL carrying bacterial isolates.

Among ESBL carrying *E*. *coli*, 20 randomly selected isolates were sequence typed resulting in 11 different STs (Table 3). The most common STs were ST 38 (five isolates) and ST 69 (five isolates) and six isolates belonged to the ST 10 clonal complex.

**Table 3:** *E*. *coli* sequence types (ST), clonal complex and ESBL genotype(s) in isolated *E*. *coli* from Franklin's gulls in Canada.

Isolate ID	ST-type	Clonal complex	ESBL genotype(s)
1	ST 167	ST10 Cplx	CTX-M 14, TEM 1
12	ST 69	ST69 Cplx	CTX-M 14
15	ST 48	ST10 Cplx	CTX-M 55, TEM 1
18	ST 69	ST69 Cplx	CTX-M 14
19	ST 69	ST69 Cplx	CTX-M 14
29	ST 69	ST69 Cplx	CTX-M 14
30	ST 38	ST138 Cplx	CTX-M 15, TEM 1
59	ST 131		CTX-M 14
68	ST 69	ST69 Cplx	CTX-M 14
88	ST 10	ST10 Cplx	CTX-M 15
101	ST 38	ST138 Cplx	CTX-M 14
108	ST 12	ST 12 cplx	CTX-M 15
122	ST 38	ST138 Cplx	CTX-M 15
130	ST 167	ST10 Cplx	CTX-M 14, TEM 52
134	ST 38	ST138 Cplx	CTX-M 15
170	ST 38	ST138 Cplx	CTX-M 15
203	ST1304		CTX-M 1
246	ST 1431		CTX-M 15
248	ST 167	ST10 Cplx	CTX-M 14, TEM 1
249	ST 617	ST10 Cplx	CTX-M 15

## Discussion

Long distance migration of birds have been implicated in the geographic spread of several pathogens [23], and are suggested to be one source for dispersal of antibiotic resistant bacteria [24]. However, formal tests of this hypothesis are lacking, in part due to the difficulties of connecting and sampling migratory populations in time and space. In an attempt to test this hypothesis, we studied resistance levels and bacterial genotype distributions in Franklin’s gulls at breeding ground in Canada and compared with similar data collected from the birds' wintering grounds in Chile [15]. Canada is a country with relatively low ESBL levels in humans [7], while Chile is a country with large antibiotic usage and high levels of resistant bacteria as ESBL [5, 15, 25]. The Franklin's gull has limited population (350,000 pair) restricted to region of North America and is an obligate migrant. If the gulls were capable of transporting resistant bacteria as part of their gut flora during migration we expected to find similarities in resistance levels and genotype distributions between the two sampled populations.

For general resistance profiles in randomly selected gull *E*. *coli* we found higher resistance levels to ampicillin and cefadroxil in Canadian gull *E*. *coli* than *E*. *coli* from Chile (Table 2). Ampicillin and tetracycline are commonly used agents and high resistance levels have been seen for these compounds in gull studies conducted elsewhere [16, 26]. Of particular interest are the very high levels of cefadroxil resistance in the randomly selected *E*. *coli* isolates, which perhaps could be hypothesized to be due to AmpC production. Plasmid-mediated AmpC are widely spread in North America and are often connected to food producing animals [27]. The high level of AmpC carrying *E*. *coli* in a non-selective culture approach is by itself alarming and warrants further studies. Apart from the compounds mentioned above, the susceptibility profiles of *E*. *coli* from Canada and Chile were rather similar, albeit based on small sample sizes.

Larger differences were seen for the analyses of ESBL-producing bacteria, where the prevalence of ESBLs was lower in Canadian Franklin's gulls (17.0%) than in Chile (30.1%). The ESBL levels in Canadian gulls was, however, significantly higher than the prevalence reported from *E*. *coli* in hospitalized patients in Canada (4.3%) in the 2009 CANWARD study [28]. Several previous studies on gulls conducted in other parts of the world have shown that levels of ESBL-producing bacteria tend to be higher in gulls compared to clinical material from the same area [22, 26, 29]; a pattern that held true also in Chile (ESBL in samples from humans 12.2%)[15].

Among the Canadian gull samples CTX-M ESBLs dominated, and the most common genotypes were *bla*_CTX-M-14_ (40.0%) and *bla*_CTX-M-15_ (30.6%). In contrast, in the gull samples from Chile, there was a strong dominance of *bla*_CTX-M-1_. In the human dataset from Canada, CTX-M dominated among the ESBL and 70.9% of the isolated CTX-M belonged to *bla*_CTX-M-15_ [7]. In other studies regarding ESBL, gull and human have shown a high degree of similarities in genotype distribution within an area/country [22, 26].

When *E*. *coli* were sequence typed, the most common STs were ST 38 and ST 69 (five isolates each), plus six isolates that belonged to the ST10 complex. These STs are also commonly reported from human bacterial collections from Canada, but differed compared to the gull isolates from Chile. In Chile, ESBL isolates showed a greater genetic variation and 31 known STs were detected, including several genotypes that were novel.

The comparisons of ESBL genotypes and *E*. *coli* STs do not provide any strong indications of long-distance dissemination between the continents. Generally, gull isolates from Canada had a lower ESBL prevalence rate, dissimilar ESBL genotypes and a more narrow set of *E*. *coli* sequence types. In Canada seven among the 11 different STs found in Canadian gulls, were also found in *E*. *coli* from Chilean gulls. Thus, the data presented here do not detect any clear signs of frequent long-distance dispersal of *E*. *coli* strains, although we acknowledge that sample sizes may have been too low and maybe the influence of the refuse dump in which the Canadian gulls were sampled could have had such a local impact so any signs of trans hemispheric exchange were diluted in this more epidemiological approach. Another plausible hypothesis is that gulls frequently acquire and loose bacteria from the environment which could explain the higher levels of ESBL in wintering gulls where there are a higher local antibiotic dissemination [25].

If persistence of particular resistant strains are short, the population of bacteria sampled may therefore more reflect the local to regional dissemination of resistant bacteria, for example from food-production animals or human waste along the migratory flyways or close to the breeding grounds. Another explanation could be that the pattern seen in Canadian is a result of clonal expansion in the population, resulting in high levels of few genotypes.

In laboratory studies it has been shown that ESBL-producing *E*. *coli* can persist in chicken microbiota for more than one month, also in lack of antibiotic pressure [30]. Furthermore, is a high transmission of *E*. *coli* strains between chickens was observed, suggesting that the population could act as a reservoir. The laboratory experiment was ended after approximately one and a half month, but the carriage pattern indicates that the retention times could be long. In the case of the Franklin's gulls there is no data on how long individual *E*. *coli* can colonize its host, and likely the frequent exposure to different isolates as part of the diet may impose competition and/or horizontal gene transfer between strains. The gulls leave their wintering grounds in March-April and reach breeding grounds in late May [14]. Thus, assuming a retention time of two months, bacteria could theoretically be disseminated to the breeding grounds. However, the likelihood for this hypothesis seems lower for than the hypothesis suggesting local dissemination, not the least since the birds were sampled at a refuse dump where presence of human-associated genotypes should be expected to occur.

The finding of similar STs between Canadian gulls and patients [9] further indicates transmission of *E*. *coli* strains between human activities and gulls in Canada. In both Canada and Chile, gulls were found to carry *E*. *coli* ST131, a well-known human-associated strain and the most common ST-type in the human material from Canada [31]. Furthermore, the ST131 found in Canadian gull carried *bla*_CTX-M-14_, and not the *bla*_CTX-M-15_ which is usually the most widely spread genotype. ST131 with *bla*_CTX-M-14_ also occurs regularly in Canadian isolates from humans[31].

The results strengthen the notion that gulls can carry high levels of ESBL and other resistance phenotype and that they thereby could act as a sentinel for dissemination of resistance to the environment. The Canadian gull samples in our study likely received a great part of their bacterial isolates from the refuse dump where they were feeding which illustrates the importance of limiting the possibility for wildlife to be contaminated with human waste products. The levels of ESBL are alarming and that the gulls in this study carry such a high prevalence makes them a possible environmental reservoir of resistant bacterial strains. Although the scoop of this article was classic ESBL, the results showing that all randomly chosen *E*. *coli* resistant to cefadroxil carried AmpC genes is highly interesting. This could indicate a high AmpC prevalence in the population that should be further studied.

More studies of the ecology, migratory pattern and feeding behavior of birds carrying ESBL-producing bacteria known to be associated with urban regions, are warranted to improve our understanding about whether wildlife play a role in the dissemination of certain antibiotic resistant bacteria.
